# Protein Interactions of the Vesicular Glutamate Transporter VGLUT1

**DOI:** 10.1371/journal.pone.0109824

**Published:** 2014-10-15

**Authors:** Magda S. Santos, Sarah M. Foss, C. Kevin Park, Susan M. Voglmaier

**Affiliations:** 1 Department of Psychiatry, University of California San Francisco, School of Medicine, San Francisco, California, United States of America; 2 Graduate Program in Cell Biology, University of California San Francisco, School of Medicine, San Francisco, California, United States of America; University of Nebraska Medical Center, United States of America

## Abstract

Exocytotic release of glutamate depends upon loading of the neurotransmitter into synaptic vesicles by vesicular glutamate transporters, VGLUTs. The major isoforms, VGLUT1 and 2, exhibit a complementary pattern of expression in synapses of the adult rodent brain that correlates with the probability of release and potential for plasticity. Indeed, expression of different VGLUT protein isoforms confers different properties of release probability. Expression of VGLUT1 or 2 protein also determines the kinetics of synaptic vesicle recycling. To identify molecular determinants that may be related to reported differences in VGLUT trafficking and glutamate release properties, we investigated some of the intrinsic differences between the two isoforms. VGLUT1 and 2 exhibit a high degree of sequence homology, but differ in their N- and C-termini. While the C-termini of VGLUT1 and 2 share a dileucine-like trafficking motif and a proline-, glutamate-, serine-, and threonine-rich PEST domain, only VGLUT1 contains two polyproline domains and a phosphorylation consensus sequence in a region of acidic amino acids. The interaction of a VGLUT1 polyproline domain with the endocytic protein endophilin recruits VGLUT1 to a fast recycling pathway. To identify *trans*-acting cellular proteins that interact with the distinct motifs found in the C-terminus of VGLUT1, we performed a series of *in vitro* biochemical screening assays using the region encompassing the polyproline motifs, phosphorylation consensus sites, and PEST domain. We identify interactors that belong to several classes of proteins that modulate cellular function, including actin cytoskeletal adaptors, ubiquitin ligases, and tyrosine kinases. The nature of these interactions suggests novel avenues to investigate the modulation of synaptic vesicle protein recycling.

## Introduction

The high frequency of neurotransmitter release observed at many synapses requires mechanisms to recycle synaptic vesicle membrane, proteins, and transmitter locally at the nerve terminal. Several mechanisms have been proposed to underlie the efficient recycling of synaptic vesicle components: classical clathrin-mediated endocytosis, budding from an endosomal intermediate, and rapid endocytosis after full fusion or kiss-and-run exocytosis [1], [2], [3], [4]. Reformation of synaptic vesicles from the plasma membrane by classical clathrin-mediated endocytosis is very similar to endocytosis occurring in non-neural cells. It requires the recruitment of a clathrin coat by adaptor proteins (APs), the acquisition of curvature mediated by endophilin, epsin and other cytosolic proteins, scission of the nascent vesicle from the plasma membrane orchestrated by dynamin, followed by uncoating triggered by the phosphatidylinositol phosphatase synaptojanin [1], [5], [6]. Dynamin and syndapin are among the “dephosphin” proteins that are regulated by a cycle of calcium-dependent dephosphorylation and phosphorylation mediated by cdk5 and GSK-3 kinases [7], [8], [9]. Thus, synaptic vesicle recycling is driven by a sequence of protein interactions and enzymatic activities [10], [11].

Models of the proposed mechanisms for synaptic vesicle recycling have assumed that the protein components of vesicles recycle together. Protein-protein interactions or retention of proteins in the cholesterol-rich synaptic vesicle membrane could cluster synaptic vesicle proteins upon exocytosis [12], [13], [14], [15]. But synaptic vesicle proteins differ in their diffusion into the plasma membrane from the site of exocytosis. While synaptotagmin, synaptophysin and VGLUT1 maintain a synaptic localization after exocytosis [16], [17], [18], [19], the v-SNARE VAMP2 rapidly diffuses away from the synapse [16], [19]. VAMP2 and synaptotagmin may also exchange with a large cell surface reservoir of these proteins [16], [20], [21], [22]. Despite differences in diffusion, some vesicle proteins appear to undergo endocytosis at the same rate [19]. In the case of VGLUT1, however, the rate of endocytosis depends on the intensity of the exocytotic stimulus and the endocytic pathway to which it is recruited, as directed by sorting signals in its protein sequence [18]. Although it is possible that synaptic vesicles retain their identity after exocytosis simply through the clustering of their components on the plasma membrane, the demonstration that synaptic vesicle proteins contain distinct sorting signals and are targeted to different endocytic pathways suggests that specific sorting of individual proteins to synaptic vesicles could be independently regulated [23], [24], [25], [26].

Three distinct vesicular glutamate transporters (VGLUT1, VGLUT2, and VGLUT3) underlie the packaging of glutamate into synaptic vesicles [27]–[38]. VGLUT1 and 2, which are responsible for the majority of glutamatergic neurotransmission, exhibit similar transport activity *in vitro*, but are largely expressed in different cell populations [39]. Expression of VGLUT1 or 2 isoforms confers differences in membrane trafficking, which may underlie differences in glutamate release properties [25], [30], [40]. VGLUTs exhibit a high level of sequence homology in the transmembrane segments that mediate glutamate transport, but diverge considerably at their cytoplasmic termini. The C-terminal domain of VGLUT1 contains several consensus sequences for protein interaction and modification that suggest these regions play a primary role in differences in membrane trafficking between the isoforms. We previously found that VGLUT1 contains multiple dileucine-like trafficking motifs that direct trafficking by distinct pathways that use different clathrin adaptor proteins [18], [25], [26]. Further, interaction of a VGLUT1 polyproline domain with the Src homology 3 (SH3) domain-containing endocytic protein endophilin targets the transporter to a faster recycling pathway during prolonged stimulation. In addition to dileucine-like and polyproline motifs, VGLUT1 contains potential ubiquitination and phosphorylation sites, suggesting that post-translational modifications may be involved in targeting and recycling of the transporter. In this work, we use VGLUT1 as a model synaptic vesicle protein to identify *cis*-acting sorting signals in the amino acid sequence and *trans*-acting factors that may direct protein sorting to specialized cellular membrane trafficking pathways involved in synaptic vesicle recycling.

## Material and Methods

### Reagents

Cell culture reagents were from Life Technologies unless otherwise noted. All other chemicals were from Sigma-Aldrich. Antibodies, suppliers and dilutions used are listed in Table 1.

**Table 1:** Antibodies used for immunoblotting and immunoprecipitation.

Antibody	Species	Source	Cat #	Dilution
Adaptin α	mouse	BD Transduction Labs	610501	1∶500
AIP4/Itch	mouse	BD Transduction Labs	611199	1∶500
β-NAP	mouse	BD Transduction Labs	610892	1∶250
CIN85	mouse	Millipore	05-731	1∶500
Endophilin 1	goat	Santa Cruz	sc-10874	1∶2000
Endophilin 3	goat	Santa Cruz	sc-8907	1∶1000
FLAG M2	mouse	Sigma	F-3165	1∶1000
Fyn	rabbit	Santa Cruz	sc-16	1∶200
HA 3F10	rat	Roche	11867423001	1∶1000; 1∶150 (IP)
HA.11	mouse	Covance	MMS-101R	1∶1000
HIP55	goat	Abcam	ab-2836	1∶500
His	mouse	Novagen/EMD Millipore	70796-3	1∶1000
IgG	rabbit	Jackson Immunoresearch	011-000-002	1 ug per reaction
Intersectin	rabbit	Regis Kelly (UCSF)	-	1∶500
*myc*	rabbit	Upstate	24165	1∶1000
nArgBP	rabbit	Pietro De Camilli (Yale)	-	1∶500
Nck	mouse	BD Transduction Labs	610099	1∶500
Nedd4	rabbit	Upstate	07-049	1∶5000
OSF1	mouse	Novus Biologicals	H00026578-B02	1∶500
Ponsin/CAP	rabbit	Upstate	06-994	1∶1000
α-spectrin	rabbit	Chemicon	AB992	1∶250
VGLUT1	goat	Santa Cruz	sc-23168/sc28940	1∶100
VGLUT1	rabbit	Robert Edwards (UCSF)	-	1∶2000
VGLUT1	Guinea pig	Chemicon	AB-5905	1∶5000
Vinexin	goat	Santa Cruz	sc-14144	1∶200
WWP1	goat	Santa Cruz	sc-11894	1∶100
WWP2	goat	Santa Cruz	sc-11897	1∶100

### Molecular biology, cell culture and transfection

Overlap extension PCR mutagenesis and site-directed PCR mutagenesis (QuikChange, Agilent Technologies) were used to introduce epitope tags and mutations, which were verified by sequencing. For expression of bacterial glutathione S-transferase (GST) fusion proteins, cDNA fragments were inserted in frame into the multiple cloning site of the pGEX-5x vector (GE Healthcare). For expression of His-tagged fusion protein (His-PP1), cDNA fragments encoding amino acids 513–549 were inserted in frame into the multiple cloning site of the Ligand Expression Vector (Panomics/Affymetrix). GST fusions of the SH3 domains of human Lyn, Fyn and Src in pGEX vectors along with full-length mouse Lyn were obtained from Clifford Lowell (UCSF). Purified GST-Lyn-SH3 protein was purchased from Panomics. *Myc*-tagged Lyn was generated by amplifying full-length mouse Lyn with primer encoding a *myc* tag followed by a four alanine linker immediately before the kinase. The resulting *myc*-Lyn was subcloned into the pcDNA1/Amp vector (Invitrogen) using standard molecular biological techniques. 3x-FLAG-tagged ubiquitin was obtained from Jeffrey Benovic (Thomas Jefferson). COS7 cells were obtained from UCSF Cell Culture Facility, grown in DME H-21 medium supplemented with 10% cosmic calf serum (Hyclone) and 1X pen/strep at 37°C in 5% CO_2_. Transient transfection by electroporation was performed as described [41]. Rat cortical neurons were isolated from embryonic day 18–20 Sprague Dawley rats (Charles River) of either sex. Prior to harvesting the embryos, pregnant female was placed in a 10.5 L acrylic chamber and euthanized with CO_2_ asphyxiation at a flow rate of 1.05–3.15 L/min followed by bilateral thoracotomy. Embryos were quickly decapitated with sharp scissors and brains were removed from the skull in ice cold HBSS. Cortex was dissected in Hibernate E (BrainBits) followed by digestion with trypsin for 5–10 min at 37°C. Dissociated neurons were transfected using a SCN Nucleofector kit (Lonza), according to manufacturer's directions [18].

### Protein purification

GST or 6x-His fusion protein expression was induced as per manufacturer's instructions with 0.5 mM IPTG for ∼4 hr at 37°C. Bacterial pellets were lysed by sonication in phosphate buffered saline (PBS) plus bacterial protease inhibitors (PIs, Sigma), sonicates cleared by centrifugation, bound to glutathione sepharose (GE) or Ni-NTA agarose beads (Qiagen), and washed extensively. His-tagged protein was eluted with 100–250 mM imidazole-containing buffer and dialyzed into 10 mM Hepes, pH 7.4, 150 mM NaCl, 4 mM EDTA and 0.005% Tween (HBS) and concentrated (Amicon, Millipore) to ∼2–10 µg/ml. Protein concentrations were measured with BCA (Thermo Scientific).

### SH3 and WW domain arrays

Purified His-PP1 was incubated with TranSignal WW (Cat #MA3030, MA3032) and TranSignal SH3 Domain Arrays (Cat. #MA3010, MA3011, MA3012, and MA3014) (Panomics/Affymetrix, now available from Gentaur, Belgium), and detected with anti-His antibody according to manufacturer's instructions [42]. The arrays were made by the manufacturer using the recombinant conserved binding sites of individual WW or SH3 domain proteins fused to GST. GST fusions are purified and immobilized onto a membrane. Each domain on the array is spotted in duplicate at 100 ng. WW domain arrays include 67 different human WW domains, whereas SH3 domain arrays include over 130 different domains.

### GST pull-down assays

GST pull-downs were performed essentially as described [18]. 10 µg GST fusion proteins bound to 10 µl beads were rotated with 250 µl brain or COS7 cell lysates (∼3 mg/ml) at room temperature (RT) for 60 min. Pelleted beads were washed with 1 ml lysis buffer and repelleted four times. Bound proteins were eluted by incubating with 10 µl reduced glutathione for 5 min at RT, then with 10 µl sample buffer for 5 min at RT. Eluted protein was subjected to SDS-PAGE and stained with Coomassie or silver, or subjected to immunoblotting. To prepare cell extracts, COS7 cells were washed, mechanically harvested and lysed in 10 mM Hepes-KOH, pH 7.4 and 150 mM NaCl containing protease inhibitors (PIs, 10 µg/ml phenylmethylsulfonyl fluoride, 2 µg/ml leupeptin, 2 µg/ml pepstatin A, 2 µg/ml E-64, 2 µg/ml aprotinin), and 2% Triton X-100 (TX-100) for 45 min on ice and the lysate cleared by centrifugation at 13,000×*g* for 15 min at 4°C. To prepare brain extracts used in the experiments of Figs. 3 and 4, rat brains were homogenized directly in 8 volumes 10 mM Hepes buffer, pH 7.4 with 0.32 M sucrose, pellet at 13,000×*g*, resuspended, solubilized in 8 volumes sucrose Hepes buffer with 2% TX-100, and pelleted at 50,000×*g* to remove cell debris. The concentration of soluble protein was assayed (Bradford reagent, BioRad) and equal amounts of protein incubated with GST fusions. Extracts from rat brain used in experiments shown in Fig. 6 were solubilized in 100 mM Tris-HCl, pH 7.5, 150 mM NaCl, 1 mM EGTA, and 1% TX-100 containing PIs (1 mg/ml E64, 2 mg/ml aprotinin, 2 mg/ml leupeptin, 2 mg/ml pepstatin, and 20 mg/ml PMSF), and sedimented at 20,000×*g* for 45 min at 4°C. The supernatant (∼400 mg total protein) was incubated with 400 µg of GST fusion proteins immobilized on glutathione sepharose beads at 4°C for 2 h. After pelleting, beads were washed and bound protein was detected by immunoblot analysis with the appropriate antibodies (see below).

### Immunoprecipitation

Cell and brain extracts were prepared as described above. For crosslinking experiments, cells were pretreated with 1 mM dithiobis (succinimidyl propionate) (DSP) for 2 h at 4°C, and quenched with 25 mM Tris [43]. Equal amounts of protein were incubated with anti-HA antibody for 1 h to overnight at 4°C, followed by incubation with Protein G sepharose beads (Roche) for 1 h. After washing 4 times with 10 volumes of lysis buffer, proteins were eluted by boiling in SDS-PAGE sample buffer, and subjected to immunoblotting.

### SDS-PAGE and immunoblotting

Samples containing 20–50 µg of protein were mixed with Laemmli sample buffer, separated by SDS-polyacrylamide gel electrophoresis (SDS-PAGE), and transferred to PVDF membrane. Membranes were blocked and immunoblotted with antibody in PBS containing 0.1% Tween and 5% nonfat dry milk, washed 3 times for 10 min, hybridized with appropriate horseradish peroxidase-coupled secondary antibodies (GE), followed by further washing, 3 times for 10 min. Detection of hybridization was performed by enhanced chemiluminescence (ECL, Thermo Scientific) and exposure of the membrane to X-ray film. Quantification of band intensities was performed using the lowest exposure that allowed detection of immunoreactive bands. ImageJ was used to determine the intensity of bands using the intensity of the respective fusion protein loaded on the same lane (revealed by Ponceau staining) to normalize the signal. Immunoblots shown are representative of at least three independent experiments.

To determine statistical significance, two-tailed t-test or one-way ANOVA followed by Bonferroni's test was performed at p<0.05 as appropriate (GraphPad Prism). Quantification data are means ± SEM of at least three independent experiments.

### ^32^P_i_ metabolic labeling

For metabolic labeling with ^32^P_i_ [44], [45], cells were washed three times in medium lacking phosphate and then incubated for 2 h at 37°C in the presence of 0.5–1.0 mCi/ml ^32^P_i_ (Perkin Elmer). After labeling, cells were washed on ice with ice-cold HBSS containing PIs and phosphatase inhibitors (50 mM NaF, 1 mM Na_3_VO_4_, 1.15 mM Na_2_MoO_4_, 2 mM imidazole, 4 mM sodium tartrate dihydrate, 2 mM β-glycerophosphate, 1 µM okadaic adic, 5 mM EDTA, 1 mM EGTA) and harvested by scraping into the same buffer; pelleted by centrifugation at 5000×*g* for 5 min at 4°C; and then resuspended by trituration in 1 ml of buffer with 2% TX-100 (homogenization buffer). After removal of the cell debris and nuclei by centrifugation at 14,000×*g* for 5 min at 4°C, SDS was added to the supernatant to a final concentration of 0.2%. For immunoprecipitation, the mixture was incubated overnight at 4°C with protein G sepharose prebound to monoclonal antibody to HA (Roche). Immune complexes were washed four times in homogenization buffer and resuspended in 2x sample buffer and the proteins separated by SDS-PAGE. Gels were fixed, dried and subjected to autoradiography.

### Ethics Statement

All animal studies were conducted in accordance with the policies and approval from the Institutional Animal Care and Use Committee for the University of California, San Francisco (Institutional PHS Assurance #A3400-01; USDA Customer #9199, Registration #93-R-0440; AAALAC Accreditation #001084).

## Results

### VGLUT C-terminal sequence domains

**Figure 1:**
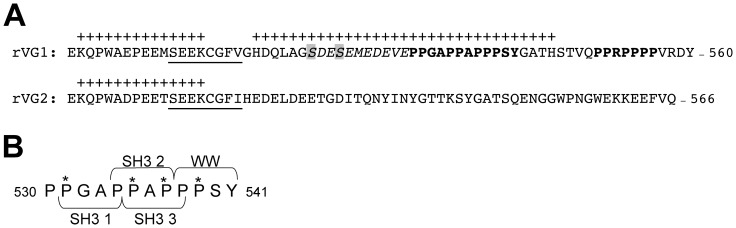
Comparison of the C-termini of rat VGLUT1 and 2. (**A**) VGLUT1 and VGLUT2 both contain an acidic dileucine-like internalization motif (underlined) and two lysine residues on either side of a potential PEST ubiquitination domain (+). VGLUT1 contains two PP domains (bold) not found in VGLUT2. VGLUT1, but not VGLUT2, also contains a region of acidic amino acids with a CK2 phosphorylation consensus sequence, **S/T**-D/E-X-D/E/pS, (italics) containing two serine residues (shaded). In addition, the VGLUT1 acidic domain and PP1 together fit the consensus for a second PEST domain (+). (**B**) VGLUT1 PP1 contains three sequences that fit the consensus for SH3 protein interaction domains (PXXP) and one for a WW protein interaction domain (PPXY). Starred proline residues are mutated singly to alanine (P531A, P537A, P535A, P539A) to individually disrupt SH3 1, 2, or 3 (PXXP), or WW (PPXY) binding. The mutation P534A + P535A disrupts all three SH3 binding domains (see Fig. 4B).

VGLUT1 and 2 exhibit a high degree of sequence homology, but diverge at their cytoplasmic termini, suggesting that these regions may mediate differences in trafficking between the two isoforms [18], [25], [30]. The C-termini of VGLUT1 and VGLUT2 both contain a potential dileucine-like internalization motif (Fig. 1A underlined) consisting of two hydrophobic amino acids with acidic residues at –4 or –5 upstream, which are thought to mediate trafficking via clathrin adaptor proteins [46], [47], [48]. VGLUT1 and 2 also both contain two lysine residues on either side of a sequence rich in proline, glutamic acid, serine and threonine residues (PEST) (Fig. 1A, +). A web-based prediction program (PESTfind) identifies a second PEST domain in VGLUT1 (Fig. 1A). PEST domains can direct ubiquitination or calpain cleavage. VGLUT2 has been shown to undergo calpain cleavage under excitotoxic conditions [49]. The C-terminus of VGLUT1 also contains two polyproline domains not present in VGLUT2 (PP1 and PP2, Fig. 1A, bold). PP1 and PP2 each contain three sequences which fit the consensus for SH3 protein interaction domains (PXXP) [50]. PP1 also contains a consensus for a WW protein interaction domain (PPXY; Fig. 1B) [51]. We have previously shown that interaction of PP2 with endophilins accelerates VGLUT1 recycling, in a manner dependent on the dileucine-like trafficking motif also present in the C-terminus [18]. The proximal C-terminus of VGLUT1 also contains an acidic region with potential phosphorylation sites (SDESEMEDEVE, Fig. 1A, italics) that fits the consensus for casein kinase 2 (CK2) phosphorylation of serines 519 and 522, as identified by NetPhosK (CK2 consensus sequence **S/T**-D/E-X-D/E/pS). The serine residue immediately upstream of the VGLUT1 acidic dileucine-like motif (S504) is identified by NetPhosK as a potential substrate for CK1 and CK2. Although the sequence around S504 (EPEEMSEE) does not fit the canonical consensus sequence for CK1 or 2 (CK1 consensus sequence S/T(p)-X_2-3_-**S/T**), non-canonical substrates include sequences containing many negatively charged amino acids [52], [53]. In addition, the sequence SYGAT is identical in all three VGLUT isoforms, and S540 is a predicted GSK-3 substrate, fitting the consensus sequence **S/T**-X-X-X-S/T(p). The presence of these motifs suggests that the VGLUT1 C-terminus could organize protein interactions to drive trafficking.

To identify *trans*-acting cellular proteins that interact with the distinct motifs found in the C-terminus of VGLUT1, we performed a series of biochemical screening assays using the amino acid residues 513–549 of the rat VGLUT1 sequence. This region encompasses the first polyproline motif, the cluster of acidic amino acids containing consensus phosphorylation sites, and the PEST domains. The first polyproline domain contains consensus sequences for SH3 and WW domain interactions (Fig. 1B). Mutation of individual proline residues to alanine were used to selectively disrupt the consensus sequences of each of the three SH3 domain-binding motifs and the WW domain-binding motif independently (Fig. 1B, asterisks). Mutation P534A + P535A disrupts all three SH3 domain-binding motifs.

### Protein interaction arrays

**Figure 2:**
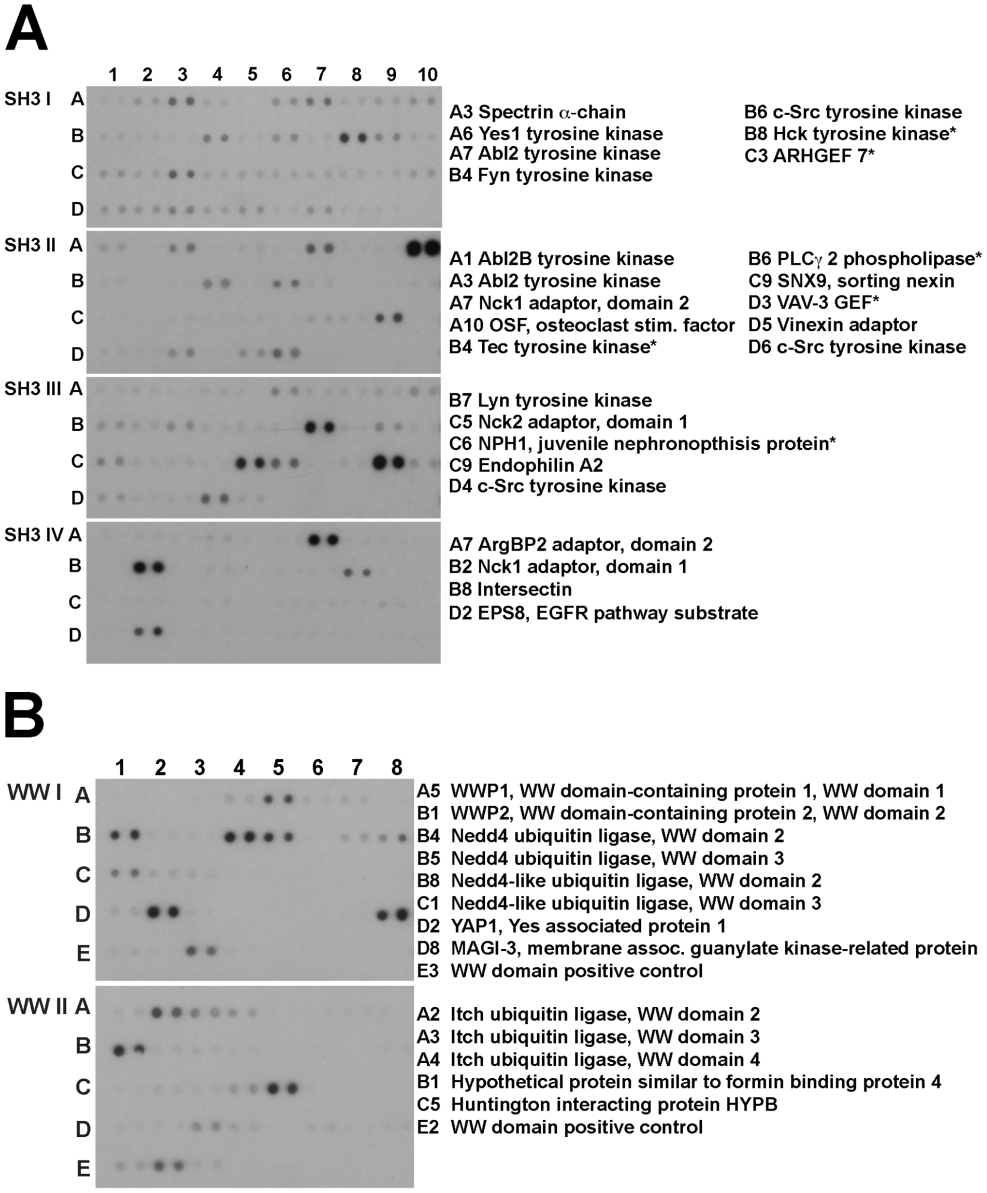
Interaction of the VGLUT1 C-terminus with SH3 and WW protein domains. GST fusions of (**A**) SH3 and (**B**) WW domains from proteins spotted in duplicate on membranes were incubated with soluble extracts from bacteria expressing a His-tagged VGLUT1 C-terminal peptide (amino acids 513-549). After washing, the bound His-VGLUT1 C-terminus was recognized by anti-His antibody coupled to horseradish peroxidase and detected by enhanced chemiluminescence. Proteins expressed at low levels in brain and those with an established function unrelated to trafficking or neurotransmitter transport are marked with an asterisk, and were excluded from further analysis.

Our yeast two-hybrid screen using the entire VGLUT1 C-terminus had previously identified the SH3 domain-containing endophilins as interactors at the second PP domain, but did not identify any other interacting proteins [18]. To identify proteins interacting with VGLUT1 PP1, SH3 and WW domain arrays (Panomics/Affymetrix) were screened using a His-tagged VGLUT1 fusion protein encompassing amino acids 513–549. The arrays cover the majority of identified SH3 and WW domains found in the human genome. Membranes spotted in duplicate with GST fusions of SH3 and WW domains from more than 150 proteins were incubated with bacterial extract containing the tagged protein and washed extensively. Bound protein was detected using antibody to the His tag (Fig. 2). Several proteins that bound His-VGLUT1 PP1 fell into three general categories—tyrosine kinases, cytoskeletal adaptors, and ubiquitin ligases. The SH3 domain-containing proteins identified include multiple Src family tyrosine kinases (Fyn, Lyn, Src, Hck); and scaffolding/adaptor proteins (e.g. Nck1, Nck2, intersectin, ArgBP2, sorting nexin 9), and endophilin (Fig. 2A). WW domain-containing proteins identified in the screen include several E3 ubiquitin ligases (e.g. Nedd4, AIP4/Itch, WWP1, WWP2; Fig. 2B). Proteins expressed at low levels in brain and those with an established function unrelated to trafficking or neurotransmitter transport were excluded from further analysis.

### Biochemical analysis of SH3 domain-containing proteins

**Figure 3:**
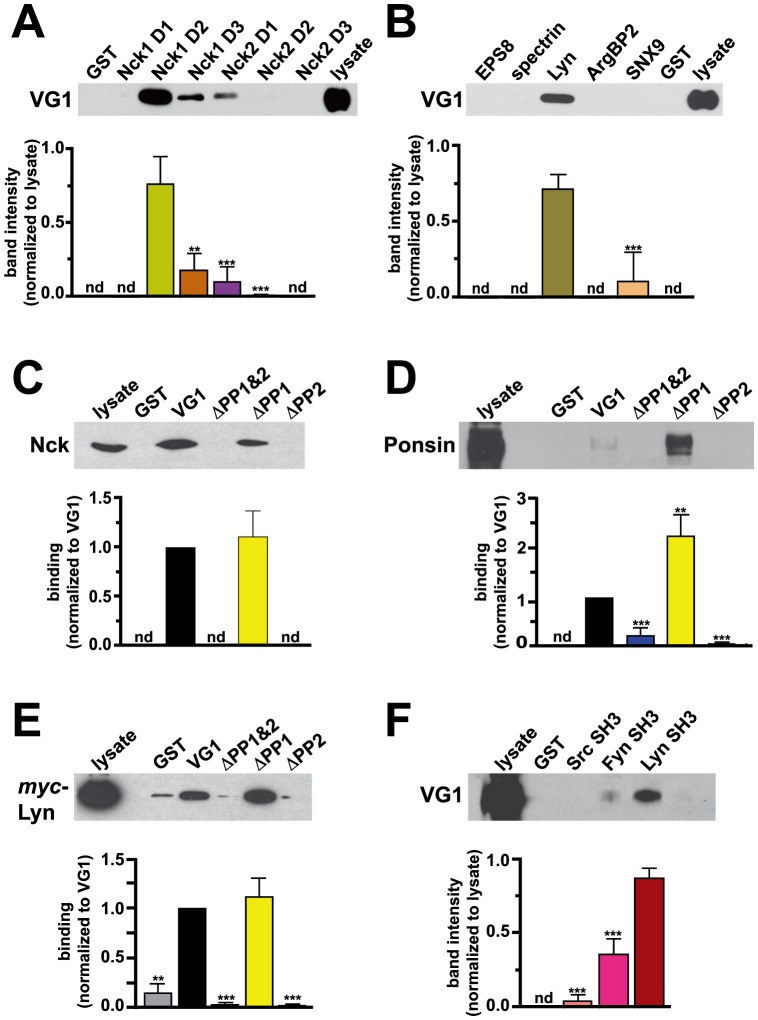
VGLUT1 interacts with SH3 domain-containing proteins *in vitro*. (**A, B**) GST or GST fusions of SH3 domains from proteins identified in the array screen were incubated with rat brain extracts. Bound VGLUT1 was detected with specific antibody, quantified and expressed in arbitrary units (a.u.). (**A**) The three SH3 domains of Nck1 and 2 (D1-3) were screened independently. Interaction with VGLUT1 is strongest for the second SH3 domain of Nck1 (Nck1 D1 not detected; Nck1 D2, 0.7635±0.1104 a.u.; Nck1 D3, 0.1833±0.0649 a.u.; Nck2 D1, 0.1031±0.0595; Nck2 D2, 0.00976±0.00564 a.u.; Nck2 D3 not detected). (**B**) The SH3 domain of Lyn pulls down VGLUT1 from rat brain lysate (0.7182±0.0987 a.u.). No binding of VGLUT1 to the SH3 domains of EPS8, spectrin, or ArgBP2 was detected. Weak binding to SNX9 was detected in only one experiment (0.1107±0.1917 a.u.). (**C, D**) Rat brain extracts were incubated with GST or GST fusions of the VGLUT1 C-terminus (VG1), or VGLUT1 lacking both PP domains (ΔPP1&2), the first PP (ΔPP1) or second (ΔPP2). Both Nck (C) and ponsin (D) bound specifically to VG1 and ΔPP1, but not to ΔPP1&2 or ΔPP2 (Nck binding to ΔPP1: 1.114±0.261 a.u. Ponsin binding to: ΔPP1&2, 0.2249±0.1682 a.u.; ΔPP1, 2.243±0.447 a.u.; ΔPP2, 0.06198±0.03914 a.u.). (**E**) Extracts from COS7 cells transfected with *myc*-Lyn were incubated with GST or GST fusions of VGLUT1 as in (C, D). Binding to Lyn was detected with antibody to *myc* (ΔPP1&2, 0.03682±0.02458 a.u., ΔPP1, 1.119±0.189 a.u.; ΔPP2, 0.02823±0.01619 a.u.). (**F**) Rat brain extracts were incubated with GST or GST fusions of the SH3 domains of the kinases Src, Fyn, or Lyn. Immunoblots probed with antibody to VGLUT1 indicate specific binding to Lyn (0.8767±0.0644 a.u.) and significantly less to Fyn (0.3622±0.1034). Band intensities were quantified using ImageJ and normalized to lysate (A, B, F) or VG1 band (C, D, E). nd: not detected. Top panels show representative immunoblots, lower panels show the averaged quantification of band intensity from at least three independent experiments. **p<0.01, ***p<0.001, one-way ANOVA with Bonferroni's post-test.

To test for *in vitro* interaction of proteins identified in the SH3 array screen, we performed GST pull-down assays with candidate proteins that were detected above background in the array screen, and fit the criteria of a) at least modest brain expression and b) a subcellular localization or function consistent with interaction with VGLUT1. Detergent-solubilized rat brain extracts were incubated with GST fusions of SH3 domains (Panomics/Affymetrix) bound to glutathione sepharose beads. Proteins bound to the beads after washing were detected by immunoblotting with an antibody to VGLUT1 (Chemicon). Using this assay, we detect binding of VGLUT1 to distinct domains of the actin cytoskeletal adaptor Nck isoforms 1 and 2 (Fig. 3A). The three SH3 domains of the two isoforms of Nck (D1-3) were screened independently. Interaction with VGLUT1 is strongest in this assay for the second SH3 domain of Nck1 (Fig. 3A, Nck1 D2). We also detect interaction of VGLUT1 with the SH3 domain of Lyn, a protein tyrosine kinase (Fig. 3B). No binding of VGLUT1 to other proteins identified in the initial screen, EPS8, spectrin, ArgBP2 or SNX9 is detected by this method (Fig. 3B).

To determine the site of interaction in VGLUT1, we used GST fusions of either the entire C-terminal cytoplasmic tail of VGLUT1 (VG1) and VGLUT1 lacking both PP domains (ΔPP1&2), the first PP (ΔPP1) or second (ΔPP2), to pull down candidate interactors. Detergent-solubilized rat brain extracts were incubated with GST fusions bound to glutathione sepharose, washed and detected by immunoblotting with antibodies to the SH3 domain-containing proteins Nck (BD Biosciences) and ponsin/c-Cbl interacting protein CAP (Upstate). Ponsin is a cytoskeletal adaptor of the sorbin homology (SoHo) family, which was reported to interact with VGLUT1 in a yeast two-hybrid screen [54]. Nck and ponsin from brain extracts bind to the GST fusion of the entire C-terminus of VGLUT1 or ΔPP1, but not to ΔPP1&2 or ΔPP2 (Fig. 3C,D). Similarly, the tyrosine kinase Lyn, from extracts of COS7 cells transfected with *myc*-tagged Lyn, binds preferentially to entire C-terminus of VGLUT1 and ΔPP1, but not to ΔPP1&2 or ΔPP2 (Fig. 3E). This suggests specific binding between PP2 and an SH3 domain of Nck, ponsin, and Lyn. To test the specificity of VGLUT1 binding to tyrosine kinases, we performed GST pull-downs using fusions of kinases that were detected in the array blots, and expressed in brain. VGLUT1 from brain extracts binds more strongly to GST-Lyn (0.8767±0.0644 a.u.) than GST-Fyn (0.3622±0.1034 a.u.) *in vitro* (Fig. 3F). No specific binding to GST-Src was detected (Fig. 3F). No binding of VGLUT1 to intersectin, CIN85, HIP55, osteoclast simulating factor (OSF) or vinexin was detected by GST pull-down assays followed by immunoblotting with specific antibodies (data not shown).

### Biochemical analysis of WW domain containing proteins

**Figure 4:**
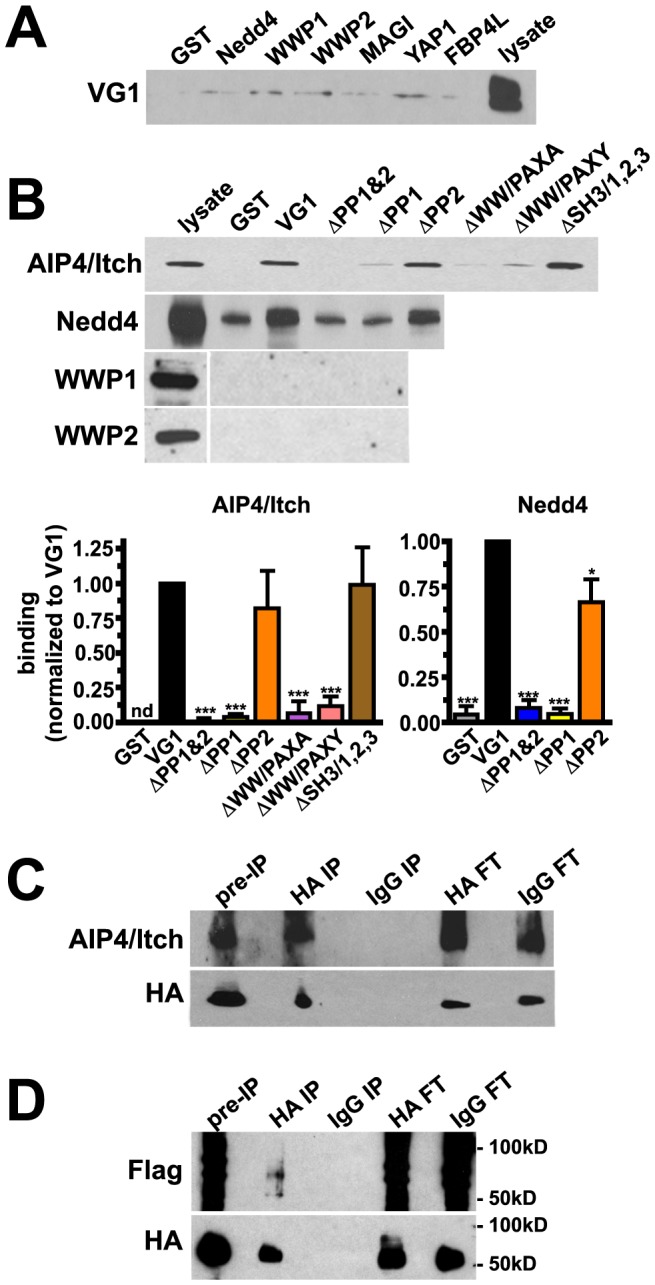
VGLUT1 interacts with the E3 ubiquitin ligases AIP4/Itch and Nedd4. (**A**) Rat brain extracts were incubated with GST or GST fusions of WW domains from proteins identified in the array screen. No specific binding to VGLUT1 was detected. (**B**) GST fusions of the VGLUT1 C-terminus (VG1), or VGLUT1 mutants lacking polyproline domains (ΔPP1, ΔPP2, ΔPP1&2), or point mutants disrupting the WW domain-binding consensus sequence (ΔWW/PAXA, ΔWW/PAXY), or all three SH3 consensus sequences in PP1 (ΔSH3/1,2,3) were incubated with rat brain lysates. Immunoblots were probed with antibody to AIP4/Itch, Nedd4, WWP1, or WWP2. Specific binding of AIP4/Itch to VGLUT1 is significantly reduced in the absence of PP1 or by disruption of the WW domain (ΔPP1&2: 0.00622±0.02337 a.u.; ΔPP1, 0.04012±0.02316 a.u.; ΔPP2, 0.8217±0.2670 a.u.; ΔWW/PAXA, 0.06691±0.08685 a.u.; ΔWW/PAXY, 0.1194±0.0668 a.u.; ΔSH3/1,2,3, 0.9896±0.2678 a.u.). Deletion of PP1 also abrogates binding of Nedd4 to VGLUT1 (ΔPP1&2, 0.08213±0.04285 a.u.; ΔPP1, 0.04586±0.03081 a.u.; ΔPP2, 0.6636±0.1280 a.u.). Band intensities were normalized to VG1. nd: not detected. Top panels show representative immunoblots, lower panels show the averaged quantification of band intensity from at least three independent experiments. *p<0.05, ***p<0.001, one-way ANOVA with Bonferroni's post-test. (**C**) Cultured rat cortical neurons transfected with HA-VGLUT1 and AIP4/Itch, were incubated with DSP crosslinker and immunoprecipitated with rat anti-HA antibodies. AIP4/Itch specifically co-immunoprecipitates with HA antibody. HA-VGLUT1 was detected with mouse anti-HA antibody. (**D**) Cultured rat cortical neurons transfected with HA-VGLUT1, 3x-FLAG-ubiquitin, and AIP4/Itch, were immunoprecipitated with rat anti-HA antibodies as in (C). Immunoprecipitates were probed with FLAG antibody to detect ubiquitination. Two bands of approximately 58 and 74 kD were specifically recognized by antibody to FLAG when immunoprecipitation was carried out with antibody to HA, but not IgG. Mouse anti-HA antibody was used to detect HA-VGLUT1. FT: flow through.

GST fusions of the WW domains of several proteins identified on the arrays did not bind VGLUT1 from brain extracts (Fig. 4A). However, GST-VGLUT1 specifically pulls down the HECT domain E3 ubiquitin ligase Atrophin Interacting Protein AIP4/Itch from brain extracts (Fig. 4B). This interaction is greatly decreased by deletion of PP1 (ΔPP1 and ΔPP1&2). Moreover, AIP4/Itch appears to specifically interact with the WW domain consensus binding site, PPXY, at the end of PP1. Disruption of the WW domain-binding consensus sequence (PPXY) by either mutating the proline residue at 538 and a tyrosine residue at 541 to alanine (ΔWW/PAXA) or the proline 538 residue alone (ΔWW/PAXY) reduces binding. In contrast, a GST fusion of VGLUT1 which disrupts all three SH3 consensus sequences by mutating proline residues 534 and 535 to alanine (ΔSH3/1,2,3), is able to bind AIP4/Itch *in vitro* (Fig. 4B). Since the closely related E3 Nedd4 is highly expressed in brain we also tested whether this protein interacts with VGLUT1. Indeed, Nedd4 is also pulled down by GST-VGLUT1 and GST-VGLUT1ΔPP2, but not GST, GST-VGLUT1ΔPP1 and GST-VGLUT1ΔPP1&2 (Fig. 4B). Two other HECT domain E3 ligases, WWP1 and 2, were not pulled down by GST-VGLUT1 (Fig. 4B). Taken together, this suggests both AIP4/Itch and Nedd4 specifically bind at the WW domain-binding consensus sequence in the first polyproline domain in the VGLUT1 C-terminus.

To determine whether VGLUT1 interacts with AIP4/Itch in cells, we co-immunoprecipitated AIP4/Itch and HA-VGLUT1. Cultured rat cortical neurons were transfected with HA-VGLUT1 and AIP4/Itch and incubated with the cross-linking agent dithiobis(succinimidyl proprionate) (DSP) [43]. Detergent extracts were immunoprecipitated with HA or IgG control antibody, and immunoblotted with antibody to AIP4/Itch. AIP4/Itch was specifically co-immunoprecipitated with antibody to HA, but not control IgG (Fig. 4C). Therefore, the interaction of AIP4/Itch and VGLUT1 occurs in cells. To determine whether VGLUT1 is ubiquitinated in neurons, we transfected rat cortical neurons with HA-VGLUT1, AIP4/Itch, and 3x-FLAG-tagged ubiquitin and immunoprecipitated with HA antibody or control IgG. Immunoprecipitates were probed with FLAG antibody to detect ubiquitination. Two bands of approximately 58 and 74 kD were recognized by antibody to FLAG when immunoprecipitation was carried out with antibody to HA, but not IgG (Fig. 4D). Thus, HA-VGLUT1 is ubiquitinated under these conditions.

### Phosphorylation of VGLUT1

**Figure 5:**
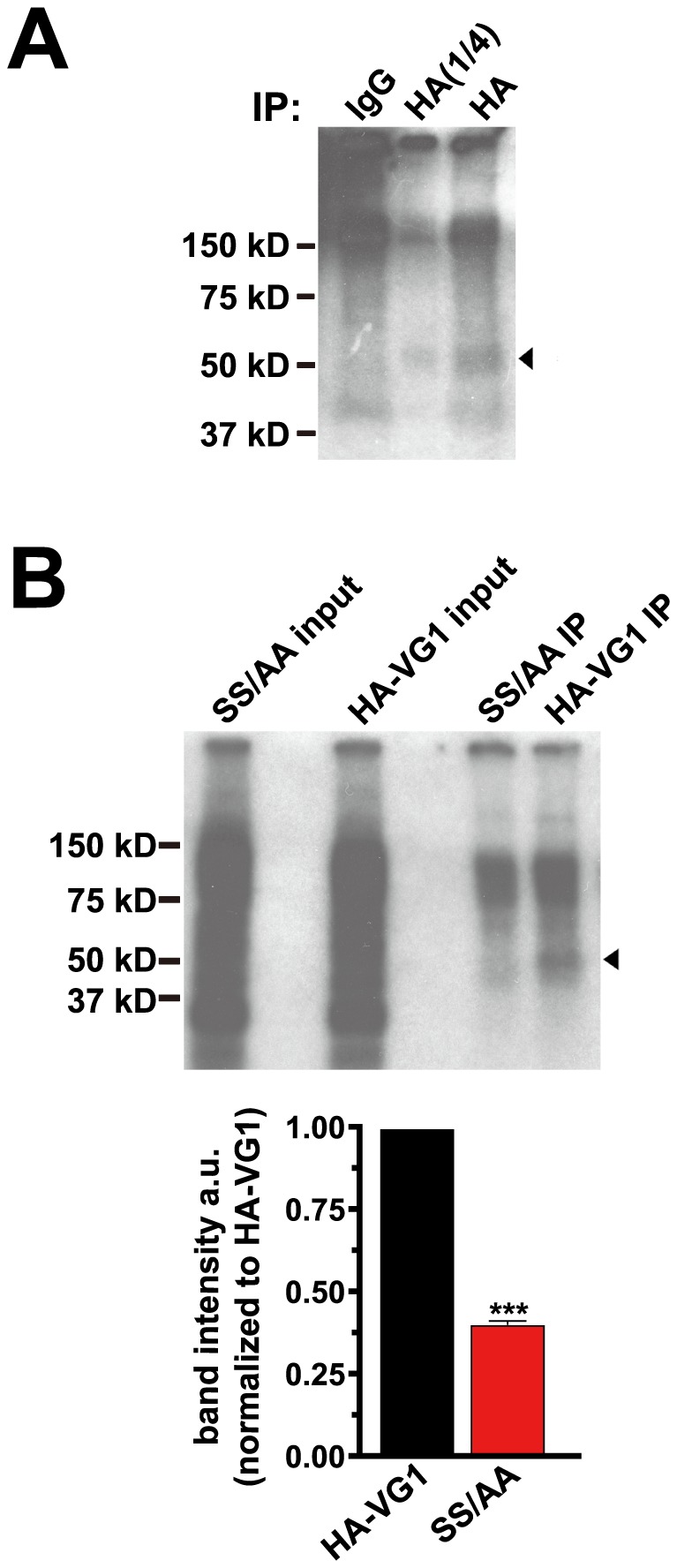
VGLUT1 phosphorylation. (**A**) Cultured rat cortical neurons transfected with HA-VGLUT1 were incubated with ^32^P_i_ for 4 h prior to immunoprecipitation with rat anti-HA antibody or rat IgG, and detected by autoradiography. A radiolabeled band of approximately 52 kD (arrowhead) was immunoprecipitated specifically with rat HA antibody, but not IgG. One lane with 1/4 amount of input, HA(1/4), was also loaded on the gel for clarity. (**B**) Rat cortical neurons transfected with HA-VGLUT1 or a mutant substituting serines 519 and 522 with alanine (SS/AA) were radiolabeled and immunoprecipitated with antibody to HA. Decreased radiolabeling of a 52 kD band (arrowhead) is noted in the SS/AA mutant (0.4028±0.0131 a.u.). Top panel shows a representative immunoblot, lower panels shows the averaged quantification of band intensities from three independent experiments. ***p<0.0001, two-tailed t-test.

The C-terminus of VGLUT1 contains a cluster of acidic amino acids that includes a consensus sequence for serine phosphorylation (NetPhos 2.0) (Fig. 1). Like the PP domains, this motif is conserved in mammalian VGLUT1 homologs, but not in VGLUT2 or 3. This sequence is similar to acidic motifs found in several membrane proteins, including the vesicular monoamine transporter, VMAT2, the epithelial sodium transporter (ENaC), the endoprotease furin, vesicle associated membrane protein 4 (VAMP4), transient receptor potential polycystin-2 channel (TRPP2), and aquaporin 4 (AQ4). Trafficking of some of these proteins is influenced by CK2-mediated serine phosphorylation [44], [55]–[60]. In the case of aquaporin 4, CK2 phosphorylation regulates its sequential binding to AP-2 to mediate endocytosis, and then to AP-3 to mediate post-endosomal trafficking [57]. Additional phosphorylation motifs may be present in VGLUT1. Indeed, we have recently demonstrated that a negatively charged residue in the vesicular GABA transporter upstream of the dileucine-like motif can modulate trafficking [26]. The analogous residue in rat VGLUT1 (S504) also fits the consensus sequence for CK2 phosphorylation (NetPhos 2.0). In addition, the serine residue in the _540_SYGAT_544_ sequence conserved in VGLUT1, -2, and -3 is also a potential phosphorylation site (NetPhos 2.0), although these were not tested here. To determine whether VGLUT1 is phosphorylated, we used ^32^P_i_ to metabolically label cultured rat cortical neurons transfected with HA-VGLUT1 at 37°C, followed by immunoprecipitation of VGLUT1 with antibody to HA (Roche) in the presence of phosphatase inhibitors, and autoradiography [44], [45]. A ^32^P_i_ labeled band approximately the predicted size of VGLUT1 is immunoprecipitated with antibody to HA, but not IgG (Fig. 5A). Substitution of serines 519 and 522 by alanine (SS/AA) within the acidic cluster decreases phosphorylation by ∼60% (Fig. 5B). Alanine mutagenesis does not completely abrogate phosphorylation, consistent with possible additional phosphorylation sites in the VGLUT1 C-terminus.

**Figure 6:**
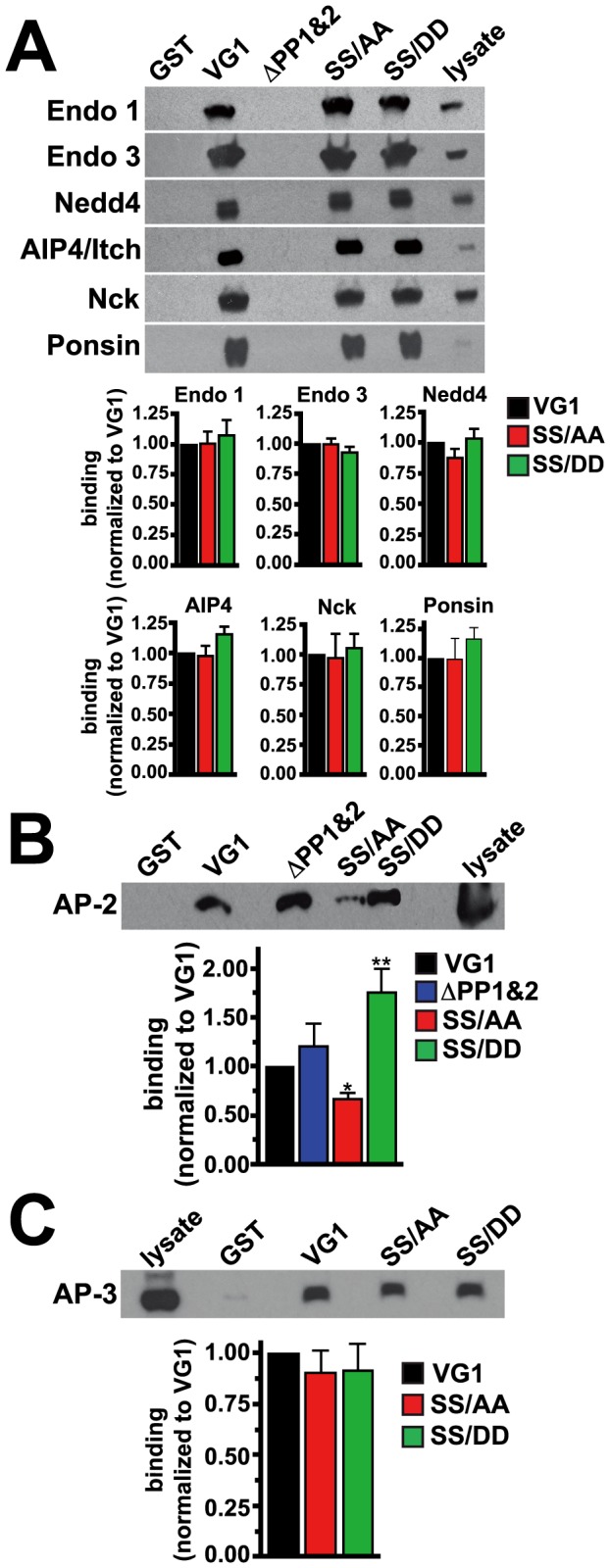
Modulation of protein interactions by phosphomimetic mutations in VGLUT1. GST pull-down assays were performed by incubating rat brain extracts with GST, or GST fusions of the WT VGLUT1 C-terminus (VG1), or mutants deleting both polyproline domains (ΔPP1&2), or mimicking the unphosphorylated (SS/AA) or phosphorylated state (SS/DD). (**A**) Bound proteins were detected by immunoblotting with antibodies against endophilin 1, endophilin 3, Nedd4, AIP4/Itch, Nck, and ponsin. Phosphomimetic mutations did not affect binding compared to VG1. Deletion of the polyproline motifs (ΔPP1&2) prevents binding of the polyproline domain interacting proteins. (**B**) Bound proteins were detected by immunoblotting with antibody against AP-2. The phosphomimetic SS/DD mutation promotes increased binding of VGLUT1 to AP-2 (1.761±0.2422 a.u.), while SS/AA mutation decreases binding of VGLUT1 to AP-2 (0.6745±0.0668 a.u.). (**C**) Bound proteins were detected by immunoblotting with antibody against AP-3. Binding of VGLUT1 to AP-3 is unaffected by serine mutations. Top panels show representative immunoblots, bottom panels show the averaged quantification of band intensity from at least three independent experiments. *p<0.05, **p<0.01, one-way ANOVA with Bonferroni's post-test.

To gain more insight into possible downstream effects of VGLUT1 phosphorylation, we performed GST pull-down experiments utilizing VGLUT1 C-terminal mutants in which serines 519 and 522 were replaced with alanine (SS/AA) or aspartate (SS/DD) to mimic the dephosphorylated and phosyphorylated states, respectively. GST fusions of wild type and mutant VGLUT1 C-terminus were bound to glutathione beads, incubated with rat brain homogenate, and analyzed by immunoblotting with antibodies to the proteins that interact at the polyproline domains. Binding to endophilins, Nedd4, AIP4/Itch, Nck, and ponsin was not affect by either of the serine mutations (Fig. 6A).

We have recently shown that binding of the clathrin adaptor protein AP-2 at the dileucine-like motif is important for VGLUT1 recycling in neurons [25]. To determine whether phosphorylation could regulate interaction of the VGLUT1 C-terminus with AP-2, we investigated whether mimicking phosphorylation of serines 519 and 522 affects binding of AP-2 and VGLUT1. As expected, GST-VGLUT1 specifically pulls down AP-2 (Fig. 6B). Interestingly, mutation to alanine (SS/AA), which mimics a dephosphorylated state, reduces this interaction. Conversely, mimicking the phosphorylated state by substitution of aspartate for the same serines (SS/DD) increases this interaction (Fig. 6B). We also tested whether serine mutations affect binding to AP-3, which has a role in synaptic vesicle recycling under conditions that trigger activity-dependent bulk endocytosis [18], [61]. In contrast to AP-2, binding of AP-3 to VGLUT1 is not affected by mutation of serines 519 and 522 (Fig. 6C). Deletion of both polyproline domains (ΔPP1&2) prevents binding of the polyproline domain interacting proteins, but not AP-2, which binds at the upstream dileucine-like motif _504_SEEKCGFV_511_ [25]. Thus, while binding of protein interactors at the polyproline domains is insensitive to phosphomimetic mutations of serines 519 and 522, binding of AP-2 is modulated by phosphomimetic mutations in VGLUT1.

## Discussion

In this work, we investigated consensus sequences for protein interaction and post-translational modification contained in the cytoplasmic C-terminal tail of VGLUT1, paying particular attention to the domains that are conserved in mammals, but differentiate this transporter from the other VGLUT isoforms. Through a series of screening and binding assays we uncovered a remarkable network of interactors belonging to several classes of protein modulators of cellular function. The results show that VGLUT1 interacts *in vitro* with actin cytoskeletal adaptor proteins, a tyrosine kinase, and ubiquitin ligases. The results further show that VGLUT1 can undergo ubiquitination and phosphorylation. Moreover, phosphorylation may regulate protein interactions of VGLUT1. These findings can drive further investigation of how VGLUT1 interacts with specialized cell biological mechanisms to direct synaptic vesicle protein recycling.

In protein arrays and GST pull-down assays, VGLUT1 PP2 interacts with an SH3 domain of Nck, an actin cytoskeletal adaptor containing one SH2 and three SH3 domains [62]. Through its SH3 domain, Nck can recruit proline-rich proteins to the plasma membrane or to multiprotein complexes found either in the cytoplasm or in association with the actin cytoskeleton. Nck activates actin polymerization [62], [63]. Ponsin/CAP was also identified as a VGLUT1 interactor in this study, as well as in a previous yeast two-hybrid screen [54]. Ponsin contains a sorbin homology domain and three C-terminal SH3 domains. Ponsin, along with ArgBP2 and vinexin, belongs to the SoHo family of proteins that regulate actin-dependent processes [64], [65]. Ponsin binds dynamin and promotes the formation of tubules decorated with actin [66]. The effects of actin disruption on synaptic vesicle recycling have been somewhat contradictory. However, there is evidence that actin is important in scaffolding of synaptic vesicles [67], [68], [69], their mobilization from synaptic vesicle pools [70]–[75], endocytosis after spontaneous release [76], ultrafast endocytosis milliseconds after exocytosis [3], and bulk endocytosis [77]–[80]. In addition, Nck could act as a scaffold to recruit other SH3 domain-containing proteins. SH3 protein interacting with Nck, 90 kDa (SPIN90) is a Nck binding protein that also interacts with dynamin and syndapin, and regulates synaptic vesicle endocytosis [81]. Investigation of the functional consequences of VGLUT1 interaction with Nck or ponsin may help clarify the role of actin in synaptic vesicle recycling, or other aspects of VGLUT1 function.

Here we also find that VGLUT1 PP2 specifically binds the tyrosine kinase Lyn. A role for Lyn in membrane protein trafficking remains unknown. The sequences around the two tyrosine residues in the VGLUT1 C-terminus are not identified as strong phosphorylation consensus motifs by a prediction program. It is possible that Lyn could exert an effect by phosphorylating other proteins involved in recycling. Tyrosine phosphorylation of synaptophysin and synapsin by Src may regulate some properties of synaptic strength [82], [83]. Interestingly, Lyn has been shown to modulate dopamine release with effects on alcohol reward [84]. Notably, endophilin, Nck, ponsin, and Lyn all bind at PP2, an arginine-rich polyproline domain. It is possible that these proteins compete for binding with each other, perhaps modulated by the phosphorylation state of the transporter. Alternatively, different populations of the transporter may bind a different cohort of proteins. Further investigation will distinguish among these possibilities.

Our screen did not uncover SH3 domain-containing proteins that bind to PP1. Instead, we discovered that VGLUT1 binds WW domain-containing ubiquitin ligases at a PPXY motif in PP1. Nedd4 and AIP4/Itch are HECT family E3 ubiquitin ligases each containing three or four WW domains, a Ca^2+^-dependent lipid binding C2 domain, and a HECT catalytic domain [85]. Nedd4-mediated ubiquitination has been shown to regulate endocytosis of the sodium channel ENaC [86], and internalization and lysosomal trafficking of AMPARs and TrkA [87], [88]. The closely related AIP4/Itch also interacts with PP1 *in vitro*. Deletion of AIP4/Itch in mice is associated with severe immune and inflammatory defects due to T cell receptor mistargeting [89]. However, the endosomally localized ubiquitin ligase AIP4/Itch is also highly expressed in neurons [90]. AIP4/Itch has been shown to interact with and ubiquitinate endophilin, which binds at PP2 of VGLUT1 [91]. Scaffolding of endophilin and ubiquitin ligase homologs signals endocytosis of several membrane proteins, including transporters, in mammals and yeast [92], [93], [94], [95]. The C2 domain present in Nedd4 or AIP/Itch could serve to coordinate scaffolding at the membrane with changes in calcium levels.

Two predicted PEST sequences in the cytoplasmic C-terminus of VGLUT1 could direct ubiquitination [49]. Immunoprecipitation experiments indicate that HA-VGLUT1 undergoes ubiquitination. Two sizes of ubiquitinated VGLUT1 bands could correspond to a mono- and a polyubiquitinated species. The conserved PEST sequence in VGLUT2 directs calpain cleavage of the transporter under excitotoxic conditions, but VGLUT1 is not cleaved by calpain [49]. The ubiquitination of VGLUT1 could suggest the potential for regulation of protein levels by degradation. Ubiquitination may also signal endocytosis of the transporter. These mechanisms could be similar to the post-endocytic sorting of receptors between recycling and degradative pathways [96]. Regulation of VGLUT1 degradation and trafficking has the potential to influence quantal size or the amount of transporter in different synaptic vesicle pools. In addition, phosphorylation of PEST sequences can influence ubiquitination and proteolysis [97]–[101]. In fact, we found evidence for phosphorylation of VGLUT1.

Calcium-regulated cycles of protein dephosphorylation and re-phosphorylation are important regulators of synaptic vesicle recycling and pool size at the presynaptic terminal [7], [79], [102], [103], [104]. Phosphorylation may also affect protein interactions [56], [57], [99], [105], [106], [107]. To assess a potential role of phosphorylation on the interaction of VGLUT1 with other proteins, we used site-directed mutagenesis to replace identified residues with either alanine to mimic the unphosphorylated state of serines 519 and 522, or aspartate to mimic phosphorylation. We determined that these mutations affect the ability of GST-VGLUT1 to bind AP-2, but not AP-3. AP-2 is thought to be the main adaptor protein functioning at the plasma membrane to internalize synaptic vesicle protein cargoes. However, the alternate adaptors AP-1 and AP-3 have been shown to be involved in synaptic vesicle formation from endosome-like structures [61], [108], [109]. The difference in the modulation of AP-2 and AP-3 binding *in vitro* by serine mutation is consistent with distinct roles for the alternate adaptors for in VGLUT1 recycling. These serines are in a cluster of acidic amino acids in the C-terminus of VGLUT1 that, like the PP domains, is conserved in mammalian VGLUT1 homologs. This sequence is also similar to acidic motifs found in several related membrane proteins, including some whose trafficking are influenced by CK2-mediated serine phosphorylation [55]–[60]. The vesicular GABA transporter VGAT and the vesicular monoamine transporter VMAT2 are phosphorylated, but non-neuronal VMAT1 is not, suggesting phosphorylation as a specific regulatory mechanism for some vesicular transporters [44], [110].

VGLUT1 contains unique domains that may reflect specialized mechanisms for regulation of its recycling, which could underlie the differences in physiological responses between neurons expressing VGLUT1 and the closely related VGLUT2. In addition to their important role in glutamate storage, VGLUTs serve as a model to understand how individual synaptic vesicle proteins recycle at the nerve terminal. In this work we investigated the VGLUT1 interactome. We identified several classes of interactors and post-translational modifications that suggest novel modes of regulation of synaptic vesicle protein recycling. Further studies will elucidate the physiological role of these modulators including the effects on neurotransmitter release. The data presented here provides a framework to understand how unique sorting sequences target individual synaptic vesicle proteins to pathways with different rates or destinations. Regulation of these mechanisms may in turn influence synaptic transmission.
